# Learning from Cochrane systematic reviews: what improvements do these suggest for the design of trials?

**DOI:** 10.12688/f1000research.22635.2

**Published:** 2020-06-25

**Authors:** Stefania Pirosca, Mike Clarke, Shaun Treweek

**Affiliations:** 1 School of Medicine, Medical Sciences and Nutrition, University of Aberdeen, Aberdeen, UK; 2 Northern Ireland Methodology Hub, Queen’s University Belfast, Belfast, UK; 3 Health Services Research Unit, University of Aberdeen, Aberdeen, UK

**Keywords:** Randomised controlled trial, Trial methodology, Systematic reviews, Schizophrenia, Multiple sclerosis.

## Abstract

**Background: **Many randomised trials have serious methodological flaws that fatally undermine their results, which makes the research wasteful. This is of concern for many, including those doing systematic reviews that include trials. Cochrane systematic reviews have a section called ‘ *Implications for research*’, which allows authors of the review to present their conclusions on how future research might be improved. Looking at these conclusions might highlight priority areas for improvement.

**Methods: **We focused on the Cochrane Schizophrenia Review Group and the Multiple sclerosis and rare diseases of the central nervous system Review Group (the MS Review Group).  Reviews with citation dates between 2009 and 2019 were identified and the recommendations of review authors in ‘ *Implications for research’* were put into categories.

**Results: **Between 2009 and 2019 we identified 162 reviews for the Schizophrenia Review Group and 43 reviews for the MS Review Group. We created 22 categories of recommendations in total, of which 12 were common to both groups. The five most used categories were the same for both: better choice of outcomes; better choice of intervention/comparator; longer follow-up; larger sample size; use of validated scales.  Better choice of outcomes and/or intervention/comparator was recommended in over 50% of reviews. Longer follow-up and larger sample size were recommended in over a third, with use of validated scales being suggested in around a fifth of reviews. There was no obvious pattern of improvement over time for trials included in systematic reviews published by both groups.

**Conclusions: **We suggest that trialists working in these and other areas ask themselves, or are compelled to do so by others (e.g. funders), why they have chosen their outcomes, intervention and comparator, whether follow-up is long enough, if the sample size is big enough and whether the scales they choose to measure their outcomes are appropriate.

## Introduction

Randomised trials are at the heart of evidence-informed healthcare systems. Not all trials, however, are created equal. Some are excellent, others have serious methodological flaws that fatally undermine their results and make the research wasteful ^1– 3^. This is of particular importance for the systematic reviews that should be used to underpin decision making in health care, and systematic reviewers often identify relevant issues and suggest ways to improve the quality of future trials. Cochrane is an international organisation that aims to provide high-quality information to support health decisions by systematically reviewing research, especially from randomised trials to investigate the effects of healthcare interventions. It is organized across more than 50 Cochrane Review Groups, each of which looks after a particular area of health.

Cochrane pays great attention to the methodological quality of both the reviews and the studies they include ^4^. Cochrane systematic reviews all have a section called ‘ *Implications for research*’, which allows the authors of the review to present their conclusions on how future research might be improved, for example by discussing the types of interventions that need more evaluation, or the outcomes which should, or should not, be measured and reported ^5^.

The work described here looked at the ‘ *Implications for research*’ sections of reviews published by two Cochrane Review Groups between 2009 and 2019 (2019 was incomplete at the time of the work). Our aims were to:

1. Categorise the recommendations made in ‘ *Implications for research*’.
2. Explore whether there have been changes over time and between the two groups.

## Methods

We focused on the Schizophrenia Review Group and the Multiple sclerosis and rare diseases of the central nervous system Review Group (which we call the MS Review Group hereafter). The Schizophrenia Review Group was chosen because it has a long-standing interest in what is written in the ‘ *Implications for research*’ section and we expected it to have good, consistent reporting. The MS group was selected because one of us (SP) had a special interest in this clinical area.

The work was split into two stages:

1. Identify and extract the ‘ *Implications for research*’ sections of all reviews in both groups with a citation date of 2009 to 2019. All reviews are available at the following URLs:

1. Schizophrenia Review Group: https://schizophrenia.cochrane.org/topic-tree
2. MS Review Group: https://msrdcns.cochrane.org/our-review

2. Categorise the recommendations on trial design and methodology made by the review authors.

Stage 1 was straightforward because the ‘ *Implications for research*’ sections of Cochrane reviews are easily identified. Stage 2 involved reading through the text and putting the issues raised by review authors into categories. We used a simple Excel spreadsheet data extraction form for this (see Data availability). We did not try to fit the data into pre-existing categories but were guided by what came from the data. The initial categorisation was done by SP before being discussed with ST until agreement was reached. Additionally we extracted information on the number of included participants and studies, the risk of bias etc. (See Data Availability for a file containing the full list). No statistical analysis was necessary beyond counting.

## Results

For the period 2009 to 2019, we identified 162 reviews for the Schizophrenia Review Group and 43 reviews for the MS Review Group. A wide variety of interventions were covered by these reviews, including drugs, educational and behavioural interventions, and other therapies such as physical exercise and acupuncture. The median number of included studies in reviews from the Schizophrenia Group was seven (range 0 to 174); for the MS Group it was five (range 0 to 45).

**Figure 1.**
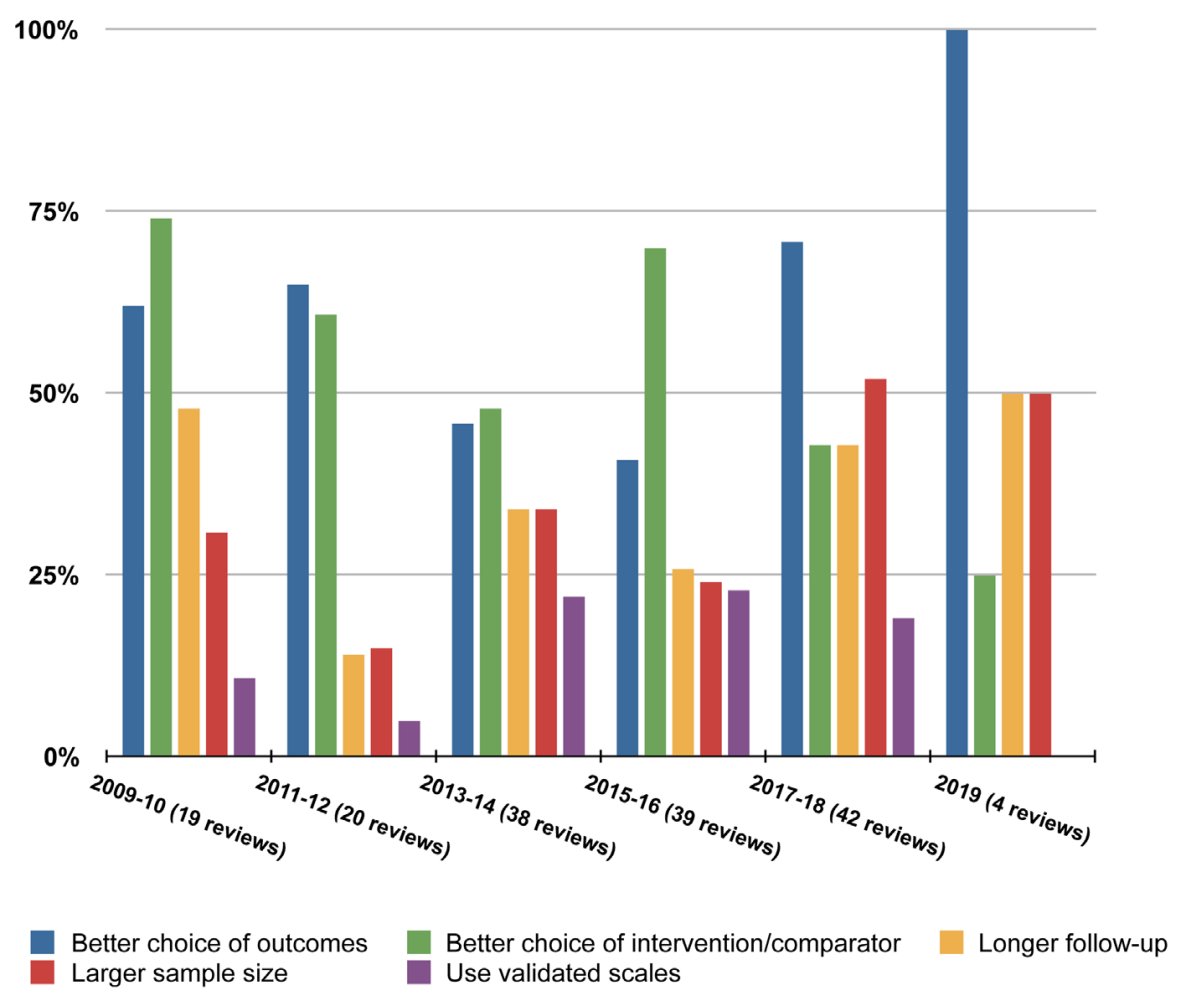
The five most used ‘ *Implications for research*’ recommendations for the Cochrane Schizophrenia Review Group 2009–2019. The percentages shown are the average over a two-year period, apart from 2019 which was incomplete at the time the work was done. While some categories are used in well over half of all review published in a given year, there is no clear pattern over time.

**Figure 2.**
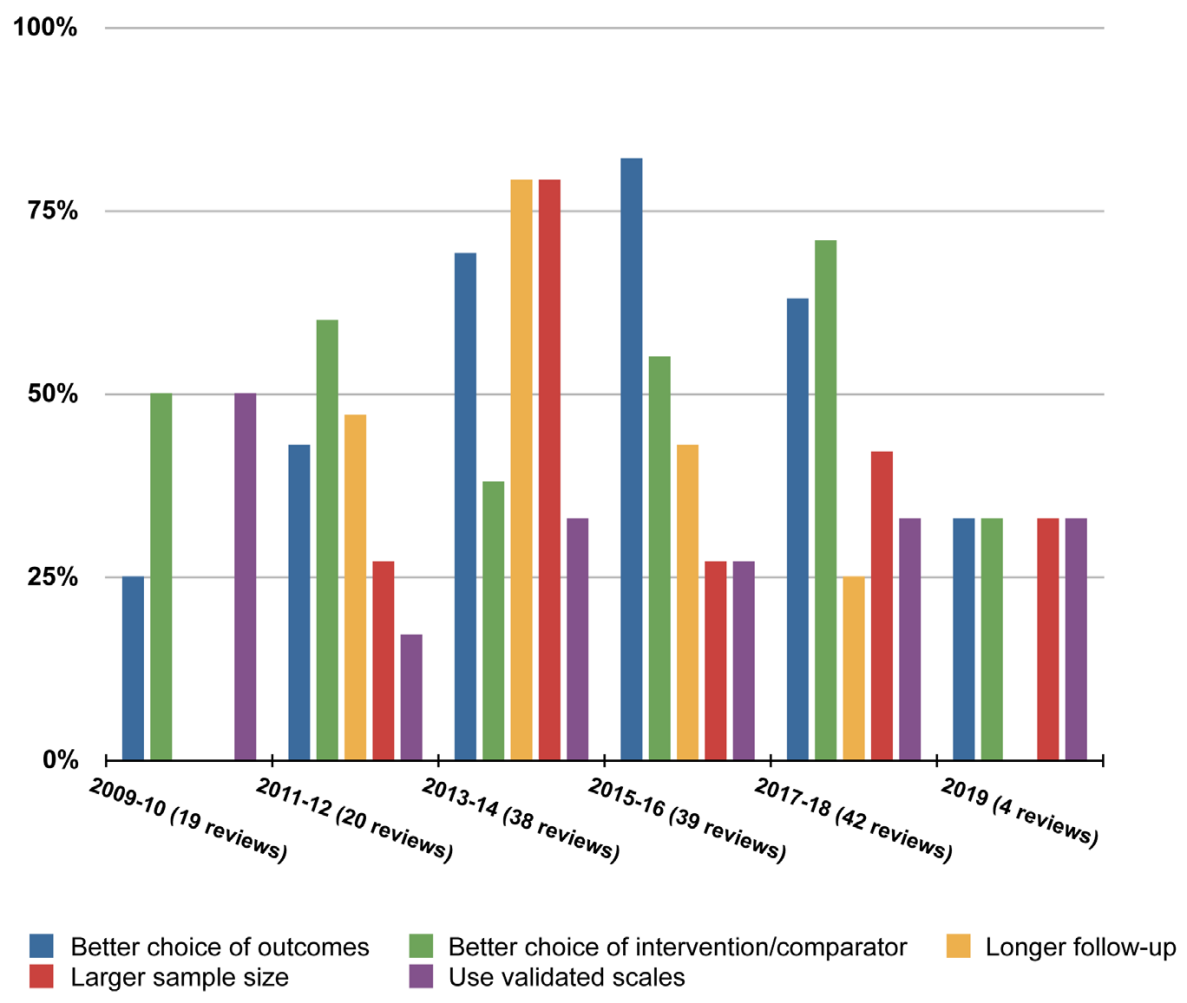
The five most used ‘ *Implications for research*’ recommendations for the Cochrane MS Review Group 2009–2019. MS=Multiple sclerosis and rare diseases of the central nervous system. The percentages shown are the average over a two-year period, apart from 2019 which was incomplete at the time the work was done. While some categories are used in well over half of all reviews published in a given year, there is no clear pattern over time. Use of ‘Better choice of outcomes’ may be increasing but review numbers are small so caution is needed in interpreting this increase.

We created 22 categories in total, of which 12 were common to both review groups ( Table 1). Six categories were unique to the Schizophrenia Review Group and four were unique to the MS Review Group. The five most used categories were the same for both groups (better choice of outcomes, better choice of intervention/comparator, longer follow-up, larger sample size and use of validated scales). However, the ranking of each category in that top five list varied from one year to the next; no category was consistently most used ( Figure 1 and Figure 2). There was no obvious pattern of improvement over time for trials included in systematic reviews published by both groups.

**Table 1.** Recommendation categories identified in review ‘Implications for research’ sections for two Cochrane Review Groups. A total of 22 categories were identified, 12 of which were common to both the Schizophrenia and Multiple Sclerosis (MS) Review Groups. The five most frequently occurring implications for research (marked in grey) were the same for both review groups.

Categories	Overall use of the category (both review groups, only categories common to both, 205 reviews) (n/%)	Use of the category in the Schizophrenia Review Group (162 reviews)	Use of the category in the MS Review Group (43 reviews)
Better outcome choice	118 (58%)	89 (55%)	29 (68%)
Better choice of future intervention and/or choice of comparator	111 (54%)	89 (55%)	22 (51%)
Longer follow-up	73 (36%)	55 (34%)	18 (42%)
Larger/sufficient sample size that has statistical power	71 (35%)	55 (34%)	16 (37%)
Use validated rating scales	39 (19%)	28 (17%)	11 (26%)
Ensure blinding (or mitigate lack of blinding where the nature of intervention makes blinding difficult)	27 (13%)	24 (15%)	3 (7%)
Better choice of eligibility criteria	19 (9%)	15 (9%)	4 (9%)
Use internationally agreed set of standardised outcomes	15 (7%)	12 (7%)	3 (7%)
Use real-world effectiveness/pragmatic design approach	10 (5%)	9 (6%)	1 (2%)
Ensure allocation is concealed	9 (4%)	7 (4%)	2 (5%)
Provide cost-effectiveness analysis	-	5 (3%)	2 (5%)
Better design	-	Not used	7 (16%)
Better methodology	-	Not used	4 (9%)
Better link to current clinical pathways	-	Not used	4 (9%)
Intention-to-treat analysis for all outcomes	-	14 (9%)	Not used
More head-to-head trials	-	Not used	3 (7%)
Do not use cross-over methodology	-	7 (4%)	Not used
More appropriate choice of trial setting	-	6 (4%)	Not used
More complete collection of participant data	-	3 (2%)	Not used
Predefine outcomes	-	3 (2%)	Not used
Include randomisation from a waiting list	-	3 (2%)	Not used

## Conclusions

There is substantial overlap in the types of recommendation made in the ‘ *Implications for research*’ sections of systematic reviews done by the Cochrane Schizophrenia and MS Review Groups. These were easier to identify in the Schizophrenia Review Group’s reviews because of their consistent approach to presenting implications for research in accordance with published guidance ^6^. Many of their reviews also routinely include a suggested design for a future trial in this section.

The five most frequently made recommendations are the same for both groups with better choice of outcomes being top of the list and used in over half the 205 reviews included in our study. Looking across the decade to 2019, there is no obvious pattern of decrease in the areas of methodology that need to be improved in trials included in systematic reviews published by both groups.

Previous research found that small, underpowered studies make up the entirety of evidence in most meta-analyses reported by Cochrane reviews ^7^. Only 35% of the reviews in our sample mentioned sample size in the Implication for research, which suggests that reviewers may be underestimating the size of this problem. The most frequent issue raised in our work was the choice of outcome, a persistent problem that led to the COMET initiative to facilitate the development of core outcome sets ^5, 8^. Cochrane reviewers need to continue to highlight trial design problems; indeed they could perhaps do so more often, especially around sample sizes.

## Limitations

It is possible that different researchers would have categorised the implications for research differently, although we did use two reviewers working independently and there was little disagreement. Selecting other Cochrane review groups may have led us to conclude that different recommendations were most common but without doing that work we have no way of knowing this.

Despite looking at just two Cochrane review groups, we believe that our findings are likely to be generalisable to other areas of health and health care but, at a minimum, a good start for the 2020s would be for researchers planning trials in schizophrenia and multiple sclerosis to ask themselves (or be compelled to do so by others, e.g. funders) the following questions:

1. Why did we choose these outcomes?

2. Why is this the right intervention and comparator?

3. Is follow-up long enough?

4. Is the sample size large enough?

5. Will validated scales be used to measure outcomes?

There are resources that can help with the above. For example COMET (http://www.comet-initiative.org) can help guide outcome choice and PRECIS-2 can help with design decisions around comparators and follow-up ^9^. Discussions with staff at clinical trial units and other research support centres are also likely to improve designs. Change is slow at present and initiatives to encourage, or force, trialists to consider these questions would be welcome, especially from funders.

## Data availability

### Underlying data

Open Science Framework: Data extracted from the Implications for Research sections of reviews from two Cochrane Review Groups. https://doi.org/10.17605/OSF.IO/XCJ7R

This project contains the following underlying data:

- Data from Implications for Research section of Cochrane MS Review Group 2009–2019.csv (Data extracted from Cochrane MS Review Group)
- Data from Implications for Research section of Cochrane Schizophrenia Review Group 2009–2019.csv (Data extracted from Cochrane Schizophrenia Review Group)

### Extended data

Open Science Framework: Data extraction form used in 'What improvements do Cochrane systematic reviewers suggest for the design of trials?'. https://doi.org/10.17605/OSF.IO/FNJVZ

This project contains the following extended data:

- Data extraction form.csv (Study data extraction form)

Open Science Framework: Full data extraction from the Implications for Research sections of reviews from two Cochrane Review Groups. https://doi.org/10.17605/OSF.IO/P9CW6

This project contains the following data:

1. Data extraction MS 12-6-2020.csv
2. Data extraction Schizophrenia 12-6-2020.csv

Data are available under the terms of the Creative Commons Attribution 4.0 International license (CC-BY 4.0).
