# Supplementary material for: Learning from Cochrane systematic reviews: what improvements do these suggest for the design of trials?
Source: F1000Res. 2020 Jun 25;9:178. Originally published 2020 Mar 11. [Version 2] doi: 10.12688/f1000research.22635.2 (PMC7194492; doi:10.12688/f1000research.22635.2)
Supplement: Peer review history

# Peer review: Reviewer response for version 2

**DOI:** 10.5256/f1000research.27618.r65530

**Published:** 2020-07-17

**Authors:** Livia Puljak

**Affiliations:** 1 Center for Evidence-Based Medicine and Health Care, Catholic University of Croatia, Zagreb, Croatia

Version 2 is great.

Is the work clearly and accurately presented and does it cite the current literature?

Yes

If applicable, is the statistical analysis and its interpretation appropriate?

Yes

Are all the source data underlying the results available to ensure full reproducibility?

Yes

Is the study design appropriate and is the work technically sound?

Yes

Are the conclusions drawn adequately supported by the results?

Yes

Are sufficient details of methods and analysis provided to allow replication by others?

Yes

Reviewer Expertise:

Methodological research, clinical epidemiology.

I confirm that I have read this submission and believe that I have an appropriate level of expertise to confirm that it is of an acceptable scientific standard.

# Peer review: Reviewer response for version 1

**DOI:** 10.5256/f1000research.24991.r62266

**Published:** 2020-04-29

**Authors:** Christian Gluud

**Affiliations:** 1 The Copenhagen Trial Unit, Copenhagen University Hospital, Copenhagen, Denmark

Pirosca and colleagues have examined the 'Implications for research' section of 205 Cochrane reviews published between 2009 to 2019 from two editorial groups. The five most used categories were the same for both editorial groups: better choice of outcomes; better choice of intervention/comparator; longer follow-up; larger sample size; use of validated scales. Better choice of outcomes and/or better choice of intervention/comparator was recommended in over half of reviews; longer follow-up and larger sample size were recommended in over a third; and use of validated scales being suggested in around a fifth of reviews. 

 I think the authors should also discus their interesting findings from the point of view: how good are the Cochrane authors? E.g. we know from numerous studies (e.g., Rebecca M Turner *et al*.) that 80% to 90% of all Cochrane reviews do not reach a fair meta-analytic sample size providing the necessary power to assess medium large and small (but still clinically relevant) intervention effects. How come then that almost 66% of reviews do not mention larger sample sizes?

Is the work clearly and accurately presented and does it cite the current literature?

Yes

If applicable, is the statistical analysis and its interpretation appropriate?

Yes

Are all the source data underlying the results available to ensure full reproducibility?

Yes

Is the study design appropriate and is the work technically sound?

Yes

Are the conclusions drawn adequately supported by the results?

Yes

Are sufficient details of methods and analysis provided to allow replication by others?

Yes

Reviewer Expertise:

Evidence based clinical practice.

I confirm that I have read this submission and believe that I have an appropriate level of expertise to confirm that it is of an acceptable scientific standard.

# Response

**Published:** 2020-06-22

**Authors:** Shaun P. Treweek

**Affiliations:** University of Aberdeen, UK

**Response to comments on F1KR00CDE F1R-VER24991-A**

**22/6/2020**

Thanks for the reviewers’ comments on our manuscript. Our responses are below.  We have made the necessary changes to the manuscript using track changes so that they can be easily seen. We have also submitted a clean version with the changes accepted.

**Reviewer #2**

- I think the authors should also discuss their interesting findings from the point of view: how good are the Cochrane authors? E.g. we know from numerous studies (e.g., Rebecca M Turner.) that 80% to 90% et al of all Cochrane reviews do not reach a fair meta analytic sample size providing the necessary power to assess medium large and small (but still clinically relevant) intervention effect. How come then that almost 66% of reviews do not mention larger sample sizes?

We have added the following text to the *‘Conclusions’* section:

Previous research found that small, underpowered studies make up the entirety of evidence in most meta-analyses reported by Cochrane reviews ^7^. Only 35% of the reviews in our sample mentioned sample size in the Implication for research, which suggests that reviewers may be underestimating the size of this problem. The most frequent issue raised in our work was the choice of outcome, a persistent problem that led to the COMET initiative to facilitate the development of core outcome sets ^5,8^.  Cochrane reviewers need to continue to highlight trial design problems; indeed they could perhaps do so more often, especially around sample sizes.

# Peer review: Reviewer response for version 1

**DOI:** 10.5256/f1000research.24991.r61192

**Published:** 2020-03-16

**Authors:** Livia Puljak

**Affiliations:** 1 Center for Evidence-Based Medicine and Health Care, Catholic University of Croatia, Zagreb, Croatia

I have reviewed with interest the manuscript titled “What improvements do Cochrane systematic reviewers suggest for the design of trials? [version 1; peer review: awaiting peer review]”. The authors report a study based on a very elegant idea, analysing content of the “Implications for research” section in selected Cochrane reviews. I would like to suggest the following minor revisions:

 Title: I would suggest revising in a way to reflect the fact that these recommendations came from Cochrane reviews. When I first read the title, I initially thought that this manuscript reports results of a survey among Cochrane authors.

 Abstract

- “Reviews with citations between 2009 and 2019 were identified” – should this be “reviews published between 2009 and 2019”?
- “We created 22 categories” – I would suggest revising “categories” into “categories of recommendations”.
- Conclusion: I am not sure that it is sufficient to recommend to trialists to “ask themselves”. The problems that were mentioned (i.e. recommendations) were consistent over the analysed period. I think it would be more meaningful to conclude that we need to analyse the effects of interventions that will force/motivate trialists to change their actions when designing clinical trials.

Methods

- It would be useful to have a section called Data extraction, to describe all the data that were extracted. For example, in the beginning of the Results, the authors have mentioned type of interventions, and number of studies included in analysed reviews, but this was not mentioned as extracted in the Methods.

Results

- Results section is rather short. I would appreciate to read more text in this section, i.e. about categories specific only to each review group analyzed. However, I understand that this is brief report, so I would not insist on this, if this is not feasible for this type of article.
- In the Discussion, the authors mentioned that some categories were “clearer”, but I am not really sure what this means. I also do not see anything mentioned about that in the Methods, and Results.
- In the Abstract, the authors wrote “ There was no obvious pattern of improvement over time for trials included in systematic reviews published by both groups”, and in the Results, the authors wrote “However, the use of these five categories varied over time for each group”. Is this supposed to be the same? The expression “varied” is very non-specific.

Discussion

- I would prefer to see this explained in more detail; I am not sure I understand completely what it means: “These were clearer in the Schizophrenia Review Group’s reviews because of their structured approach to presenting implications for research in accordance with published guidance”. What kind of “structured approach” is this?
- Is this a formal requirement, is there a reference to be used for this sentence: “Their reviews also routinely include a suggested design for a future trial in this section.”
- “cited in over half” – I would suggest to revise “mentioned (or used) in over half”.
- Conclusion statement at the end of the manuscript: the same comment as for the conclusion statement at the end of the Abstract.
- Perhaps the authors should mention some other resources that the trialists should use to improve some of these aspects, such as core outcome sets.

Is the work clearly and accurately presented and does it cite the current literature?

Yes

If applicable, is the statistical analysis and its interpretation appropriate?

Yes

Are all the source data underlying the results available to ensure full reproducibility?

Yes

Is the study design appropriate and is the work technically sound?

Yes

Are the conclusions drawn adequately supported by the results?

Yes

Are sufficient details of methods and analysis provided to allow replication by others?

Yes

Reviewer Expertise:

Methodological research, clinical epidemiology.

I confirm that I have read this submission and believe that I have an appropriate level of expertise to confirm that it is of an acceptable scientific standard.

# Response

**Published:** 2020-06-22

**Authors:** Shaun P. Treweek

**Affiliations:** University of Aberdeen, UK

**Response to comments on F1KR00CDE F1R-VER24991-A**

**22/6/2020**

Thanks for the reviewers’ comments on our manuscript. Our responses are below.  We have made the necessary changes to the manuscript using track changes so that they can be easily seen. We have also submitted a clean version with the changes accepted.

**Reviewer #1**

- Title: I would suggest revising in a way to reflect the fact that these recommendations came from Cochrane reviews. When I first read the title, I initially thought that this manuscript reports results of a survey among Cochrane authors.

We have changed the title to “Learning from Cochrane systematic reviews: what improvements do these suggest for the design of trials?”

- “Reviews with citations between 2009 and 2019 were identified” – should this be “reviews published between 2009 and 2019”?

In the first draft of the report we had ‘published’ but we have decided to change to ‘citations’ because of the way Cochrane makes its reviews available. Through ‘citation’ we mean that we examined the most recent version of any review that was first published or was updated in 2009 to 2019. This is important because reviews from before 2009 were still “published” during the decade, but were not updated in that time period.

- “We created 22 categories” – I would suggest revising “categories” into “categories of recommendations”.

Done. 

- Conclusion: I am not sure that it is sufficient to recommend to trialists to “ask themselves”. The problems that were mentioned (i.e. recommendations) were consistent over the analysed period. I think it would be more meaningful to conclude that we need to analyse the effects of interventions that will force/motivate trialists to change their actions when designing clinical trials. 

We have added a few sentences including the available resources for researchers to change their actions in the *‘Conclusions’* section:

There are resources that can help with the above. For example, COMET (http://www.comet-initiative.org) can help with outcome choice and PRECIS-2 can help with design decisions around comparators and follow-up ^9^.  Discussions with staff at clinical trial units and other research support centres are also likely to improve designs.  Change is slow at present and initiatives to encourage, or force, trialists to consider these questions would be welcome, especially from funders.

In the abstract, we have also added a few words to address this comment (addition in italics, which is also included in the main Conclusions):

We suggest that trialists working in these and other areas ask themselves, *or are compelled to do so by others (e.g. funders)*,…

- It would be useful to have a section called Data extraction, to describe all the data that were extracted. For example, in the beginning of the Results, the authors have mentioned type of interventions, and number of studies included in analysed reviews, but this was not mentioned as extracted in the Methods.

We have added a new file in the repository including all the data we extracted so that readers can see the data we extracted.  We have added the following text to the *‘Methods’* section:

Additionally we extracted information on the number of included participants and studies, the risk of bias etc. (See Data Availability for a file containing the full list).

- Results section is rather short. I would appreciate to read more text in this section, i.e. about categories specific only to each review group analyzed. However, I understand that this is brief report, so I would not insist on this, if this is not feasible for this type of article.

As this is a Brief Report, we have decided to keep the *‘Results’* section to the point and we have not repeated in words information that is in the table. We have added some extra detail though, see response to Comment #8.

- In the Discussion, the authors mentioned that some categories were “clearer”, but I am not really sure what this means. I also do not see anything mentioned about that in the Methods, and Results.

We have replaced this text with:

However, the ranking of each category in that top five list varied from one year to the next; no category was consistently most used (Figures 1 and 2).  There was no obvious pattern of improvement over time for trials included in systematic reviews published by both groups.  

- In the Abstract, the authors wrote “ There was no obvious pattern of improvement over time for trials included in systematic reviews published by both groups”, and in the Results, the authors wrote “However, the use of these five categories varied over time for each group”. Is this supposed to be the same? The expression “varied” is very non-specific.  

The two sentences refer to different things: the one in the abstract to general improvement over time, the one in Results to the position of a given category in the top five from one year to the next.  We have modified the text, see response to Comment #7.

- I would prefer to see this explained in more detail; I am not sure I understand completely what it means: “These were clearer in the Schizophrenia Review Group’s reviews because of their structured approach to presenting implications for research in accordance with published guidance”. What kind of “structured approach” is this?

Consistency was the key advantage and we have changed the wording of this sentence accordingly:

These were easier to identify in the Schizophrenia Review Group’s reviews because of their consistent approach to presenting implications for research in accordance with published guidance ^6^

- Is this a formal requirement, is there a reference to be used for this sentence: “Their reviews also routinely include a suggested design for a future trial in this section.”

We are not aware of this being a formal requirement, or of a reference we can point readers to.  We have modified the sentence so that it now comes across as an observation rather than review group policy:

Many of their reviews also include a suggested design for a future trial in this section.

- “cited in over half” – I would suggest to revise “mentioned (or used) in over half”.

We have changed to ‘used in over half’. 

- Conclusion statement at the end of the manuscript: the same comment as for the conclusion statement at the end of the Abstract.

We have addressed this issue. See response to comment 4. 

- Perhaps the authors should mention some other resources that the trialists should use to improve some of these aspects, such as core outcome sets.

See response to Comment #4.
